# On relative simple Heffter spaces

**DOI:** 10.1007/s10801-026-01573-0

**Published:** 2026-08-17

**Authors:** Laura Johnson, Lorenzo Mella, Anita Pasotti

**Affiliations:** 1 School of Mathematics, University of Bristol, Bristol, BS8 1UG United Kingdom; 2 DICATAM - Sez. Matematica, Università degli Studi di Brescia, Via Branze 43, I-25123 Brescia, Italy

**Keywords:** Heffter system, Partial linear space, Orthogonal cycle decomposition

## Abstract

In this paper, we introduce the concept of a relative Heffter space which simultaneously generalizes those of relative Heffter arrays and Heffter spaces. Given a subgroup *J* of an abelian group *G*, a relative Heffter space is a resolvable partial linear space whose points form a half-set of G\J and whose blocks are all zero-sum in *G*. Here we present two infinite families of relative Heffter spaces satisfying the additional condition of being simple. As a consequence, we get new results on globally simple relative Heffter arrays, on mutually orthogonal cycle decompositions and on biembeddings of cyclic cycle decompositions of the complete multipartite graph into an orientable surface.

## Introduction

The concept of a Heffter space has been recently introduced in [8] as a generalization of the well-known notion of a Heffter array, see [1]. In this paper we consider *relative* Heffter spaces which are a natural generalization of relative Heffter arrays introduced in [15] and also of Heffter spaces. Firstly, we recall some necessary concepts and notation.

Given an additive group *G* of order 2v+t and a subgroup *J* of *G* of order *t*, a *half-set* of G\J is a size *v* subset *V* of *G* such that $V\cup (-V)=G\setminus J$. When *J* is the trivial subgroup, that is when t=1, one simply says that *V* is a half-set of *G*.

### Definition 1.1

Let *G* be an abelian group of order 2nk+t, *J* be a subgroup of *G* of order *t* and *V* be a half-set of G\J. An $(nk,k)_t$ *relative Heffter system* on *V* is a partition of *V* into *n* zero-sum parts, called *blocks*, of size *k*.

When t=1, the subscript is omitted and we acquire a classical (*nk*, *k*) Heffter system.

### Definition 1.2

Two $(nk,k)_t$ relative Heffter systems $\mathcal {P}$ and $\mathcal {Q}$ on the same half-set are *orthogonal* if every block of $\mathcal {P}$ intersects every block of $\mathcal {Q}$ in at most one element.

### Example 1.3

The set $V = \{-1,2,3,-4,5,-6,-7,8,-10,11,12,-13,14,-15,-16,17,-19,20,21,-22\}$ is a half-set of $\mathbb {Z}_{45}\backslash {J}$, where *J* is the subgroup of $\mathbb {Z}_{45}$ of order 5. The following sets, $\mathcal {P}$ and $\mathcal {Q}$, are $(20,4)_5$ relative Heffter systems on *V*.

**Figure:**
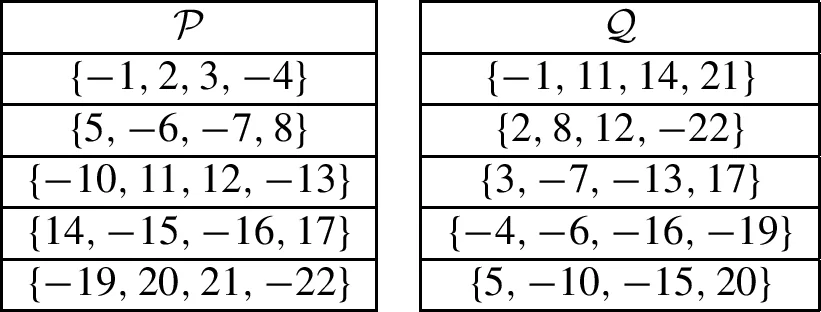

In fact, the sets $\mathcal {P}$ and $\mathcal {Q}$ are mutually orthogonal $(20,4)_{5}$ relative Heffter systems since their blocks intersect in at most one element.

An $(nk,k;r)_t$ *relative Heffter space* is nothing more than a set of *r* mutually orthogonal $(nk,k)_t$ relative Heffter systems. In order to give a more formal definition we have to recall some concepts from classical design theory, see [3]. A *partial linear space* (PLS, for short) is a pair $(V, \mathcal {B})$ where *V* is a set of points and $\mathcal {B}$ is a set of nonempty subsets (called *blocks* or *lines*) of *V* with the property that any two distinct points are contained together in at most one block. A PLS where every two distinct points are contained in exactly one block is said to be a *linear space*. The *degree* of a point of a PLS is the number of blocks containing that point. A PLS has *degree* *r* if all its points have the same degree *r*. A *parallel class* of a PLS is a set of blocks partitioning the point set. A PLS is said to be *resolvable* if there exists a partition of the block set (called *resolution*) into parallel classes. By a *resolved* PLS we mean a resolvable PLS together with a specific resolution of it. In this paper, we will focus on resolvable PLSs in which all blocks have the same size, these are known as *configurations*, but note that all definitions in this paper can also be given for partial linear spaces that are not configurations. Clearly, a configuration with *v* points, constant block size *k* and degree *r* has necessarily $b=\frac{vr}{k}$ blocks.

### Definition 1.4

Given an abelian group *G* and a subgroup *J* of *G*, a *Heffter space over* *G* *relative to* *J* is a resolved partial linear space whose parallel classes are mutually orthogonal relative Heffter systems on a half-set of G\J.

When *G* is a cyclic group, we speak of a *cyclic* relative Heffter space and of *cyclic* relative Heffter systems. If *J* is the trivial subgroup we find the concept of a Heffter space introduced in [8].

Note that the degree of the space is just the number of mutually orthogonal relative Heffter systems of the space. The aim of research in this area is to construct (relative) Heffter spaces with largest possible degree. In [7, 8] the authors construct infinite classes of Heffter spaces with an arbitrary large degree. Other very recent results on Heffter spaces have been obtained in [6]. When the degree of the space is 2, namely when we have only 2 orthogonal (relative) Heffter systems, the (relative) Heffter space is in fact a (relative) Heffter array (see [1, 15]). More precisely, a $(nk,k;2)_t$ relative Heffter space is nothing but a $\textrm{H}_t(n;k)$ relative Heffter array which can be formally defined as follows.

### Definition 1.5

Let *G* be an abelian group of order 2nk+t and let *J* be a subgroup of *G* of order *t*. A $\textrm{H}_t(n;k)$ *Heffter array over* *G* *relative to* *J* is an array of order *n* with elements in *G* such that:

- each row and each column has exactly *k* filled cells;
- the entries form a half-set of G\J;
- every row and every column is zero-sum in *G*.

For instance the Heffter systems $\mathcal {P}$ and $\mathcal {Q}$ in Example 1.3 are the parallel classes of a $(20,4;2)_5$ relative Heffter space and can be displayed by the following $\textrm{H}_5(5;4)$ Heffter array over $\mathbb {Z}_{45}$.

$$\begin{array}{|r|r|r|r|r|r|r|} \hline -1 &  2 &  3 &  -4 &  \\ \hline &  8 &  -7 &  -6 &  5 \\ \hline 14 &  &  17 &  -16 &  -15 \\ \hline 21 &  -22 &  &  -19 &  20 \\ \hline 11 &  12 &  -13 &  &  -10 \\ \hline \end{array}$$

If t=1, the subscript notation is omitted, and we find again the concept of a square Heffter array introduced by Archdeacon in [1], whose existence has been completely established, in fact it is known that there exists a $\textrm{H}(n;k)$ if and only if $n\ge k \ge 3$, see [2, 12, 19]. On the other hand, when t>1 the existence problem is still largely open, partial results can be found in [15, 17, 22, 24, 25]. We point out that in the definitions proposed in [1, 15] the elements of the array belong to a cyclic group, but more generally one can consider a Heffter array with entries in an abelian group, as done in Definition 1.5. For variants and generalizations of classical Heffter arrays see [26]. Furthermore, it is worth noting that (relative) Heffter spaces fall within the class of the so-called *additive combinatorial designs* which were introduced in [5] as a generalization of the concept of *additive* 2-*design* defined in the seminal paper [11].

As explained in [8], a Heffter space is the more interesting the closer it is to a linear space, a good parameter to measure this distance is the so-called density of the space. The *density* δ of a $(nk,k;r)_t$ relative Heffter space is defined as the density of the collinear graph associated to the space and, reasoning as in [8], one can find that $\delta = \frac{r(k-1)}{nk-1}$. The space is linear if and only if δ=1. Here we focus on relative Heffter spaces having high density and which are *simple*. One of the motivations for studying simple relative Heffter spaces is that, starting from the blocks of such a space, one can construct a set of mutually orthogonal cycle decompositions of the complete multipartite graph, as explained in Sect. 5. We say that a *k*-subset *B* of an abelian group *G* is *simple* if there exists an ordering $\{b_0,b_1,\ldots ,b_{k-1}\}$ of the elements of *B* such that the *k*-sequence of its partial sums $(c_0,c_1,\ldots ,c_{k-1})$, where $c_i = \sum \limits _{j=0}^i b_j$ for $0 \le i \le k-1$, does not have any repeated elements. We say that a relative Heffter space is *simple* if each of its blocks admits a simple ordering. Note that since the blocks of a Heffter system sum to 0, a Heffter system is simple if and only if we can order the elements of its blocks in such a way that no subsequence sums to 0.

In this paper we firstly present some preliminary results which allow us to construct, in Sect. 3, two infinite classes of simple relative Heffter spaces (see Theorems 3.3 and 3.7), one of which always achieves the maximal density. Then, in Sect. 4, we get, as a consequence, two new infinite classes of relative Heffter arrays (see Theorems 4.1 and 4.4) satisfying the very strong additional condition of being globally simple. Finally, in the last section, we present new constructive results regarding sets of mutually orthogonal cyclic cycle decompositions of the complete multipartite graph and biembeddings of these decompositions into an orientable surface.

## Preliminary results

In this section, we record some preliminary results that will be used to identify relative Heffter space constructions in Sect. 3. Given two integers *a* and *b*, by [*a*, *b*] we denote the set $\{a,a+1,\ldots , b\}$ if a≤b, while [*a*, *b*] is empty if a>b. Also, given a subset *S* of $\mathbb {Z}_w$, by ∑S we denote the sum of all the elements in *S*. If *V* is a half-set of $\mathbb {Z}_w$ whose elements have zero-sum in $\mathbb {Z}$ we will say that *V* is an *integer* half-set of $\mathbb {Z}_w$. Firstly we prove an existence result on simple integer half-sets in a cyclic group.

### Proposition 2.1

Let k≥3 be an integer. Then there exists an integer half-set of $\mathbb {Z}_{2k+1}$ admitting a simple ordering.

### Proof

We divide the proof into cases, depending on the value of *k* modulo 4.

**Case** $k \equiv 1 \pmod {4}$. If k=5, it is immediate to check that $L = \{-10,-2,3,4,5\}$ is an integer half-set of $\mathbb {Z}_{11}$ admitting a simple ordering. For k≥9 consider the following half-set of $\mathbb {Z}_{2k+1}$:

$$\begin{aligned} L =&\{-1,-2,3\} \cup \left\{ 2i,-(2i+1)\mid i\in \left[ 2, \frac{k+3}{4}\right] \right\} \cup \\&\left\{ -2i,2i+1 \mid i\in \left[ \frac{k+7}{4}, \frac{k-3}{2}\right] \right\} \cup \{-(k-1),k+1\}. \end{aligned} $$

It can be easily seen that the sum of the elements in *L* is zero in $\mathbb {Z}$: the first bracket sums to zero, while the second and third brackets add to $-\frac{k-1}{4}$ and $\frac{k-9}{4}$, respectively, and the last one sums to 2. A simple ordering of *L* is then:

$$ \begin{aligned}&\left( k+1,-1,4,-5,6,-7,\dotsc , \frac{k+3}{2}, - \frac{k+5}{2}, -2,-(k-1), k-2, \right. \\&\qquad ~~~~~~~~~~~~~~~~~~~~~~~~~~~~~~~~~~~~~~~~~~~~~~~~~~~~~~~~~~~\left. -(k-3), \dotsc , -\frac{k+7}{2},3 \right) . \end{aligned} $$

Indeed, its partial sums are:

$$ \begin{aligned} (k+1,k,k+4,k-1,k+5,k-2,\dotsc , \frac{5k+11}{4}, \frac{3k+1}{4},\frac{3k-7}{4}, \\ -\frac{k+3}{4},\frac{3k-11}{4},-\frac{k-1}{4},\dotsc , -3,0), \end{aligned} $$

that are all distinct.

**Case** $k \equiv 2 \pmod {4}$. We construct the following half-set of $\mathbb {Z}_{2k+1}$:

$$ L = \left\{ 2i-1, -2i \mid i \in \left[ 1, \frac{k+2}{4}\right] \right\} \cup \left\{ -(2i-1),2i \mid i \in \left[ \frac{k+6}{4}, \frac{k}{2}-1\right] \right\} \cup \{-(k-1),k+1\}. $$

It can be easily verified that *L* has zero-sum in $\mathbb {Z}$; a simple ordering of *L* is:

$$ \left( k+1,1,-2,3,-4,\dotsc , \frac{k}{2}, -\frac{k+2}{2}, -(k-1),k-2,-(k-3),\dotsc , \frac{k+6}{2}, -\frac{k+4}{2}\right) , $$

with partial sums

$$ \left( k+1,k+2,k,k+3,k-1, \dotsc , \frac{5k+6}{4}, \frac{3k+2}{4}, \frac{-k+6}{4}, \frac{3k-2}{4}, \frac{-k+10}{4}, \dotsc , \frac{k+4}{2},0\right) $$

that are all distinct.

**Case** $k \equiv 3 \pmod {4}$. If k=3, then choose $L = \{1,2,-3\}$, where clearly any ordering of its elements is simple. Assume then that k≥7, and consider the following half-set *L* of $\mathbb {Z}_{2k+1}$:

$$ L = \{1\}\cup \left\{ 2i,-(2i+1) \mid i \in \left[ 1,\frac{k+1}{4}\right] \right\} \cup \left\{ -2i, 2i+1 \mid i \in \left[ \frac{k+5}{4}, \frac{k-1}{2}\right] \right\} . $$

It is not hard to see that *L* has zero-sum in $\mathbb {Z}$; a simple ordering of *L* is:

$$ \left( k,2,-3,4,-5,\dotsc , \frac{k+1}{2}, - \frac{k+3}{2}, -(k-1), k-2, -(k-3), \dotsc ,\frac{k+7}{2}, -\frac{k+5}{2},1 \right) $$

whose partial sums are:

$$\begin{aligned}  &   \left( k,k+2,k-1,k+3,k-2,\dotsc , \frac{5k+5}{4}, \frac{3k-1}{4}, -\frac{k-3}{4},\frac{3k-5}{4},\right. \\  &   \qquad \left. \frac{-k+7}{4},\dotsc , \frac{k+3}{2},-1,0\right) . \end{aligned}$$

**Case** $k \equiv 0 \pmod {4}$. Construct the half-set *L* of $\mathbb {Z}_{2k+1}$ defined as:

$$ L = \left\{ 2i-1, -2i \mid i \in \left[ 1,\frac{k}{4}\right] \right\} \cup \left\{ -(2i-1), 2i \mid i \in \left[ \frac{k}{4}+1, \frac{k}{2}\right] \right\} . $$

Also in this case, one can easily check that *L* has zero-sum in $\mathbb {Z}$; a simple ordering of *L* is:

$$ \left( k,1,-2,3,-4,\dotsc , -\frac{k}{2}, -(k-1),k-2, -(k-3), \dotsc , \frac{k}{2}+2, -\left( \frac{k}{2}+1\right) \right) $$

since its partial sums are:

$$ \left( k, k+1, k-1,k+2,k-2, \dotsc ,\frac{3k}{4}, -\frac{k}{4}+1,\frac{3k}{4}-1, -\frac{k}{4}+2,\dotsc , \frac{k}{2}+1, 0 \right) . $$

□

### Example 2.2

In this example we construct a simple ordering of a zero-sum half-set of $\mathbb {Z}_{2k+1}$, following the proof of Proposition 2.1, for k∈[13,16]. Note that for each of the partial sums, we have chosen a representative of each congruence class that is in the range [-k,k].

**Table:** 

*k*	Simple ordering	Partial sums
13	(14,-1,4,-5,6,-7,8,-9,-2,-12,11,-10,3)	(-13,13,-10,12,-9,11,-8,10,8,-4,7,-3,0)
14	(15,1,-2,3,-4,5,-6,7,-8,-13,12,-11,10,-9)	(-14,-13,14,-12,13,-11,12,-10,11,-2,10,-1,9,0)
15	(15,2,-3,4,-5,6,-7,8,-9,-14,13,-12,11,-10,1)	(15,-14,14,-13,13,-12,12,-11,11,-3,10,-2,9,-1,0)
16	(16,1,-2,3,-4,5,-6,7,-8,-15,14,-13,12,-11,10,-9)	(16,-16,15,-15,14,-14,13,-13,12,-3,11-2,10,-1,9,0)

Now we present two results about a particular collection of sequences. Firstly we consider the case of an odd-length sequence.

### Lemma 2.3

Let k≥3 be an odd integer. There exists $A = (a_0,\ldots ,a_{k-1})$ such that, if, for i∈[0,k-1],

we set $A_i=(\alpha _{i,0},\ldots ,\alpha _{i,k-1})$ where $\alpha _{i,j}=j\cdot a_{i+j}$ (all subscripts are considered modulo *k*), then

- $$\sum A= \sum A_0=0$$,
- $\sum A_i=k$ if *i* is odd,
- $\sum A_i=-k$ if i≥2 is even.

### Proof

Let $A = (a_0,\ldots ,a_{k-1})$ be defined as

$$a_i={\left\{ \begin{array}{ll}1 &  \mathrm{if \ } i=0,k-1\\ -2 &  \mathrm{if\ } i \mathrm{\ odd\ with\ } 1\le i\le k-2 \\ 2 &  \mathrm{if\ } i \mathrm{\ even \ with\ } 2\le i\le k-3. \end{array}\right. } $$

- Notice that the sequence *A* comprises 2 1s, $\frac{k-1}{2}$ -2’s and $\frac{k-3}{2}$ 2’s: it is therefore immediate that this sequence sums to 0. Consider now $A_0$. Since for any *j* even with j∈[2,k-3] we have $\alpha _{0,j}+\alpha _{0,j+1}=ja_j+(j+1)a_{j+1}=2j-2(j+1)=-2$, then $\sum _{j=2}^{k-2}\alpha _{0,j}=-2\frac{k-3}{2}=-k+3$. Since $\alpha _{0,0}=0$, $\alpha _{0,1}=-2$, $\alpha _{0,k-1}=k-1$, the thesis follows.
- If i=1, for *j* odd with j∈[1,k-4] we have $\alpha _{1,j}+\alpha _{1,j+1}=2j-2(j+1)=-2$, hence $\sum _{j=1}^{k-3}a_{1,j} =-2\frac{k-3}{2}=-k+3$. Since $\alpha _{1,0}=0$, $\alpha _{1,k-2}=k-2$, and $\alpha _{1,k-1}=k-1$ the thesis follows. Suppose now i≥3 odd. If *j* is odd with j∈[1,k-i-3] then $\alpha _{i,j}+\alpha _{i,j+1}=ja_{i+j} + (j+1)a_{i+j+1} = 2j-2(j+1)=-2$ since i+j is an even integer not exceeding k-3. Hence $\sum _{j=1}^{k-i-2}\alpha _{i,j}=-2\frac{k-i-2}{2}=-k+i+2$. While if *j* is odd with j∈[k-i+1,k-2] then $\alpha _{i,j}+\alpha _{i,j+1}=-2j+2(j+1)=2$ since $i+j\equiv 2\ell +1\pmod k$ with $\ell \in {[0,\frac{k-5}{2}]}$. Hence $\sum _{j=k-i+1}^{k-1}\alpha _{i,j}=2\frac{i-1}{2}=i-1$. Note also that $\alpha _{i,0}=0$, $\alpha _{i,k-i-1}=k-i-1$ and $\alpha _{i,k-i}=k-i$. Then $\sum A_i=(-k+i+2)+(i-1)+(k-i-1)+(k-i)=k$.
- Suppose now i≥2 even. If *j* is odd with j∈[1,k-i-4] then $\alpha _{i,j}+\alpha _{i,j+1}=-2j+2(j+1)=2$ since i+j is an odd integer not exceeding k-4. Hence $\sum _{j=1}^{k-i-3}\alpha _{i,j}=2\frac{k-i-3}{2}=k-i-3$. If *j* is even with j∈[k-i+1,k-3] then $\alpha _{i,j}+\alpha _{i,j+1} = -2j+2(j+1)=2$ since $i+j\equiv 2\ell +1\pmod k$ with $\ell \in {[0,\frac{k-4}{2}]}$. Hence $\sum _{j=k-i+1}^{k-2}\alpha _{i,j}=2\frac{i-2}{2}=i-2$. Note also that $\alpha _{i,0}=0$, $\alpha _{i,k-i-2}=-2(k-i-2)$, $\alpha _{i,k-i-1}=k-i-1$, $\alpha _{i,k-i}=k-i$ and $\alpha _{i,k-1}=-2(k-1)$. Then $\sum A_i=(k-i-3)-2(k-i-2)+(k-i-1)+(k-i)+(i-2)-2(k-1)=-k$.

□

### Example 2.4

Take k=7 then:

$$\begin{aligned} \begin{array}{ll} A = (1,-2,2,-2,2,-2,1) & \quad A_3 = (0,2,-4,3,4,-10,12)\\ A_0 = (0,-2,4,-6,8,-10,6) & \quad A_4 = (0,-2,2,3,-8,10,-12)\\ A_1 = (0,2,-4,6,-8,5,6) & \quad A_5 = (0,1,2,-6,8,-10,12)\\ A_2 = (0,-2,4,-6,4,5,-12) & \quad A_6 = (0,1,-4,6,-8,10,-12)\\ \end{array} \end{aligned}$$

It is immediate to check that $\sum A=\sum A_0=0$, $\sum A_1=\sum A_3=\sum A_5=7$ and $\sum A_2=\sum A_4=\sum A_6=-7$.

Now we focus on a class of sequences of even length.

### Lemma 2.5

Given an even integer k≥4, there exists $A = (a_0, \dotsc , a_{k-1})$ such that $\sum A=\sum _{i=0}^{k-1} ia_i= 0$.

### Proof

We split the proof into two cases according to the congruence class of *k* modulo 4.

Case 1: k=4ℓ for some $\ell \ge 1$. Let *A* be defined as follows

$$a_i={\left\{ \begin{array}{ll}1 &  \mathrm{if \ } i\equiv 0,3 \pmod {4},\\ -1 &  \mathrm{if\ } i\equiv 1,2 \pmod {4}. \end{array}\right. } $$

From this definition, it is immediate to verify that ∑A=0 and it results:

$$ \begin{aligned} \sum _{i=0}^{k-1} ia_i&= \sum _{j=0}^{\ell -1} (4j\,a_{4j} + (4j+1)\, a_{4j+1}+ (4j+2)\,a_{4j+2}+(4j+3)\,a_{4j+3}) \\&= \sum _{j=0}^{\ell -1} (4j - (4j+1)- (4j+2)+(4j+3)) = 0. \end{aligned} $$

Case 2: k=4ℓ+2 for some $\ell \ge 1$. Let *A* be defined as follows

$$a_i={\left\{ \begin{array}{ll} -2 &  \mathrm{if \ } i=0,\\ 2 &  \mathrm{if \ } i=1,\\ 1 &  \mathrm{if \ } i \in \{2,4\} \mathrm{\ or \ } i\equiv 1,2 \pmod {4} \mathrm{\ and \ } i\ge 6,\\ -1 & \mathrm{if \ } i \in \{3,5\} \mathrm{\ or \ } i\equiv 0,3 \pmod {4} \mathrm{\ and \ } i>6. \\ \end{array}\right. } $$

From this definition, it is immediate to verify that ∑A=0 and it results:

$$ \begin{aligned} \sum _{i=0}^{k-1} ia_i&= (0a_0+1a_1+2a_2+\dotsc +5a_5)+ \sum _{j=1}^{\ell -1} ((4j+2)a_{4j+2} \\  &\quad +(4j+3)a_{4j+3}+(4j+4)a_{4j+4}+(4j+5)a_{4j+5}) \\&= (2+2-3+4-5)+ \sum _{j=1}^{\ell -1} ((4j+2)-(4j+3)-(4j+4)+(4j+5))=0. \\ \end{aligned} $$

□

We conclude this section by covering some group theoretical results. By $\textrm{U}(\mathbb {Z}_w)$ we denote the group of units of the cyclic group $\mathbb {Z}_w$ of order *w*. Also, given $s \in \mathbb {Z}_w$, by $\langle {s}\rangle$ we mean the additive subgroup of $\mathbb {Z}_w$ generated by *s*.

### Lemma 2.6

Let $s,a \in \mathbb {Z}_w$ with $a \not \in \langle {s}\rangle$ and *w* odd. Then the additive inverse of every element of the additive coset $a + \langle {s}\rangle$ is contained within the additive coset $s-a+ \langle {s}\rangle$ and vice versa. Moreover $(a + \langle {s}\rangle ) \cap (s-a + \langle {s}\rangle ) = \emptyset$.

### Lemma 2.7

Let w=kd(2k+1), then every element of the subgroup $\langle {2k+1}\rangle$ of $\mathbb {Z}_w$ can be expressed as a unique element of the form (id+j)(2k+1), where i∈[0,k-1] and j∈[0,d-1].

### Proof

Observe that the additive subgroup $\langle {2k+1}\rangle$ of the group $\mathbb {Z}_w$ can be partitioned into *d* cosets of the smaller additive subgroup $\langle {d(2k+1)}\rangle$ of $\mathbb {Z}_w$, which has cardinality *k*. More specifically, for each j∈[0,d-1], the coset $j(2k+1) + \langle {d(2k+1)}\rangle$ of $\langle {d(2k+1)}\rangle$ is a subset $\langle {2k+1}\rangle$. Notice that every element of the additive subgroup $\langle {d(2k+1)}\rangle$ may be written as id(2k+1) for some i∈[0,k-1], therefore every element of the subgroup $\langle {2k+1}\rangle$ can be written in the form (id+j)(2k+1), where i∈[0,k-1] and j∈[0,d-1]. □

## Constructions of simple relative Heffter spaces

Now we use the results of Sect. 2 to construct two infinite classes of simple relative Heffter spaces. In both cases, the points of the space form a half-set of $\mathbb {Z}_{n(2k+1)}\setminus \langle {2k+1}\rangle$ for suitable choices of *n* and *k*. We then show that when n=k is a prime, the constructed Heffter spaces are as dense as possible.

As usual by Φ(k) we will denote Euler’s totient function of a positive integer *k*.

### Proposition 3.1

Let *n* be an odd integer and *k* be a divisor of *n*. Then there exist at least Φ(k)+1 simple cyclic $(nk,k)_n$ relative Heffter systems.

### Proof

Set n=kd and w=n(2k+1). Let $A = (a_0,\ldots ,a_{k-1})$ be the zero-sum sequence in $\mathbb {Z}_{2k+1}$ constructed in Lemma 2.3. By Proposition 2.1, there exists an integer half-set $L=\{\ell _0,\ell _1,\ldots ,\ell _{k-1}\}$ of $\mathbb {Z}_{2k+1}$ admitting a simple ordering $\omega =(\ell _0,\ell _1,\ldots ,\ell _{k-1})$. Now set $\mathcal {P}_0 = \{B_{i,j,0} \mid i\in [0,k-1], j\in [0, d-1]\}$, where

$$\begin{aligned} B_{i,j,0}&:= \{a_0(id+j)(2k+1)+\ell _0,a_1(id+j)(2k+1)\\&\quad \,+\ell _1,a_2(id+j)(2k+1)+\ell _2,\ldots ,a_{k-3}(id+j)(2k+1)\\&\quad \, +\ell _{k-3},a_{k-2}(id+j)(2k+1)\\&\quad \,+\ell _{k-2},a_{k-1}(id+j)(2k+1)+\ell _{k-1}\}. \end{aligned}$$

We will show that $\mathcal {P}_0$ is a simple Heffter system in $\mathbb {Z}_{w}$ relative to $\langle {2k+1}\rangle$. We start by proving that each block $B_{i,j,0}$ sums to zero in $\mathbb {Z}_w$. Note that

$$\begin{aligned} \sum B_{i,j,0}=\sum _{m=0}^{k-1} \left( a_m(id+j)(2k+1)+\ell _{m}\right) =(id+j)(2k+1) \sum A +\sum L=0. \end{aligned}$$

Now we prove that the blocks of $\mathcal {P}_0$ partition a half-set of $\mathbb {Z}_w\backslash \langle {2k+1}\rangle$. Notice that we can partition the set $\mathbb {Z}_w\backslash \langle {2k+1}\rangle$ into 2*k* non-trivial cosets of the additive subgroup $\langle {2k+1}\rangle$. It follows from Lemma 2.6 that for each a∈[1,k] the additive inverses of the elements contained within the coset $a + \langle {2k + 1}\rangle$ are all contained within the coset $2k+1-a + \langle {2k+1}\rangle$, therefore, if we can prove that $\mathcal {P}_0$ either contains a copy of the coset $a + \langle {2k + 1}\rangle$ or $2k+1-a + \langle {2k + 1}\rangle$ for all a∈[1,k], then it follows that the blocks of $\mathcal {P}_0$ form a partition of a half-set of $\mathbb {Z}_w\backslash \langle {2k+1}\rangle$. Since each element of *L* is either in the coset $a+\langle {2k+1}\rangle$ or $2k+1-a+\langle {2k+1}\rangle$ for each a∈[1,k], and by Lemma 2.7 each element of the coset $\langle {2k+1}\rangle$ may be uniquely expressed as (id+j)(2k+1) for some i∈[0,k-1] and j∈[0,d-1], it is immediate that $\mathcal {P}_0$ is a relative Heffter system of $\mathbb {Z}_w\backslash \langle {2k+1}\rangle$.

It remains to demonstrate that the relative Heffter system $$\mathcal {P}_0$$ is simple. To see this, let $$\omega ' = (a_0(id+j)(2k+1) + \ell _0,\ldots ,a_{k-1}(id+j)(2k+1)+\ell _{k-1})$$ be an ordering of the elements of an arbitrary block $$B_{i,j,0}$$ of $$\mathcal {P}_0$$, where i∈[0,k-1] and j∈[0,d-1]. Observe that since the ordering $$\omega = (\ell _0,\ldots ,\ell _{k-1})$$ of *L* is simple in the group $$\mathbb {Z}_{2k+1}$$, none of the subsequences of ω sum to 0 modulo 2k+1. It then follows that, since all multiples of 2k+1 reduce to 0 modulo 2k+1, the subsequences of $\omega^{\prime }$ will also not sum to 0 modulo 2k+1 and hence the subsequences of $\omega^{\prime }$ will not sum to 0 modulo kd(2k+1). It therefore follows that $$\mathcal {P}_0$$ is a simple relative Heffter system.

Now we construct other Φ(k) simple relative Heffter systems. For any $s \in U(\mathbb {Z}_{k})$ define now $\mathcal {P}_s = \{B_{i,j,s} \mid i\in [0,k-1],j\in [0,d-1] \}$ where

$$\begin{aligned} B_{i,j,s}&= \{a_{i}j(2k+1) + \ell _{i},a_{1+i}(ds+j)(2k+1) \\&\quad \,+ \ell _{1+i}, a_{2+i}(2ds+j)(2k+1) \\&\quad \,+ \ell _{2+i},\ldots ,a_{k-2+i}((k-2)ds+j)(2k+1)\\&\quad \,+\ell _{k-2+i},a_{k-1+i}((k-1)ds+j)(2k+1) +\ell _{k-1+i}\} \end{aligned}$$

and all subscripts are considered modulo *k*.

We prove that each $\mathcal {P}_s$ is a simple Heffter system in $\mathbb {Z}_{w}$ relative to $\langle {2k+1}\rangle$. Notice that the elements of each block $B_{i,j,s} \in \mathcal {P}_s$ sum to

$$\begin{aligned} \sum _{m=0}^{k-1} [a_{m+i}(mds+j)(2k+1)+\ell _{m+i}]= (2k+1)\sum _{m=0}^{k-1} a_{m+i}(mds+j)+ \sum _{m=0}^{k-1} \ell _{m+i}= \end{aligned}$$

$$\begin{aligned} (2k+1)ds\sum _{m=0}^{k-1} ma_{m+i}+j(2k+1)\sum _{m=0}^{k-1} a_{m+i}+\sum L. \end{aligned}$$

Since $\sum _{m=0}^{k-1} a_{m+i}=\sum A=0$ and ∑L=0 we have

$$\begin{aligned} \sum B_{i,j,s}=(2k+1)ds\sum _{m=0}^{k-1} ma_{m+i}. \end{aligned}$$

Notice that $\sum _{m=0}^{k-1} ma_{m+i}=\sum A_i$, where $A_i$ is the sequence defined in Lemma 2.3, is 0, *k* or -k. In each case we get $\sum B_{i,j,s}\equiv 0 \pmod {kd(2k+1)}$, that is $B_{i,j,s}$ sums to zero in $\mathbb {Z}_w$. Now we prove that for any $s \in \textrm{U}(\mathbb {Z}_{2k+1})$, the elements of the blocks of $\mathcal {P}_s$ form a half-set of $\mathbb {Z}_w\backslash \langle {2k+1}\rangle$. Previously it was demonstrated that each element of the coset $\ell _m + \langle {2k+1}\rangle$ may be uniquely expressed by $\ell _m + a_m(id+j)(2k+1)$, where $a_m \in A$ is fixed, i∈[0,k-1] and j∈[0,d-1]. Since $s \in \textrm{U}(\mathbb {Z}_{2k+1})$, it follows that each element of the coset $\ell _m + \langle {2k+1}\rangle$ can also be expressed by $\ell _m + a_m(ids+j)(2k+1)$. This means that every block $B_{i,j,s} \in \mathcal {P}_s$ contains precisely one element of each coset $\ell _m + a_m(ids+j)(2k+1)$, where $\ell _m$ is a member of the half-set *L* of $\mathbb {Z}_{2k+1}$, hence the elements of the blocks of $\mathcal {P}_s$ form a half-set of $\mathbb {Z}_w\backslash \langle {2k+1}\rangle$. Finally, by reasoning as in the case of $\mathcal {P}_0$, one can prove that for any *s* in $\textrm{U}(\mathbb {Z}_{2k+1})$ the relative Heffter system $\mathcal {P}_s$ is simple.

Since $\Phi (k)=|U(\mathbb {Z}_{2k+1})|$, we have the thesis. □

### Example 3.2

Let n=15 and k=5, then d=3, 2k+1=11 and w=165. For k=5 we have A=(1,-2,2,-2,1) and ω=(-10,-2,3,4,5). Following the proof of Proposition 3.1, we get the five simple cyclic $(75,5)_{15}$ relative Heffter systems $\mathcal {P}_0,\ldots ,\mathcal {P}_4$ listed below:

**Table:** 

	$\mathcal {P}_0$	$\mathcal {P}_1$	$\mathcal {P}_2$
$B_{0,0,s}$	$\{-10,-2,3,4,5\}$	$\{-10,-68,-30,-29,-28\}$	$\{-10,31,-63,-62,-61\}$
$B_{1,0,s}$	$\{23,-68,69,-62,38\}$	$\{-2,69,37,-61,-43\}$	$\{-2,-30,70,38,-76\}$
$B_{2,0,s}$	$\{56,31,-30,37,71\}$	$\{3,-62,71,-76,64\}$	$\{3,37,-28,23,-35\}$
$B_{3,0,s}$	$\{-76,-35,36,-29,-61\}$	$\{4,38,56,-35,-63\}$	$\{4,71,-43,-68,36\}$
$B_{4,0,s}$	$\{-43,64,-63,70,-28\}$	$\{5,23,31,36,70\}$	$\{5,56,64,69,-29\}$
$B_{0,1,s}$	$\{1,-24,25,-18,16\}$	$\{1,75,-8,-51,-17\}$	$\{1,9,-41,81,-50\}$
$B_{1,1,s}$	$\{34,75,-74,81,49\}$	$\{-24,-74,15,-50,-32\}$	$\{-24,-8,48,49,-65\}$
$B_{2,1,s}$	$\{67,9,-8,15,82\}$	$\{25,81,82,-65,42\}$	$\{25,15,-17,34,-57\}$
$B_{3,1,s}$	$\{-65,-57,58,-51,-50\}$	$\{-18,49,67,-57,-41\}$	$\{-18,82,-32,75,58\}$
$B_{4,1,s}$	$\{-32,42,-41,48,-17\}$	$\{16,34,9,58,48\}$	$\{16,67,42,-74,-51\}$
$B_{0,2,s}$	$\{12,-46,47,-40,27\}$	$\{12,53,14,-73,-6\}$	$\{12,-13,-19,59,-39\}$
$B_{1,2,s}$	$\{45,53,-52,59,60\}$	$\{-46,-52,-7,-39,-21\}$	$\{-46,14,26,60,-54\}$
$B_{2,2,s}$	$\{78,-13,14,-7,-72\}$	$\{47,59,-72,-54,20\}$	$\{47,-7,-6,45,-79\}$
$B_{3,2,s}$	$\{-54,-79,80,-73,-39\}$	$\{-40,60,78,-79,-19\}$	$\{-40,-72,-21,53,80\}$
$B_{4,2,s}$	$\{-21,20,-19,26,-6\}$	$\{27,45,-13,80,26\}$	$\{27,78,20,-52,-73\}$

**Table:** 

	$\mathcal {P}_3$	$\mathcal {P}_4$
$B_{0,0,s}$	$\{-10,-35,69,70,71\}$	$\{-10,64,36,37,38\}$
$B_{1,0,s}$	$\{-2,36,-62,-28,56\}$	$\{-2,-63,-29,71,23\}$
$B_{2,0,s}$	$\{3,-29,38,-43,31\}$	$\{3,70,-61,56,-68\}$
$B_{3,0,s}$	$\{4,-61,23,64,-30\}$	$\{4,-28,-76,31,69\}$
$B_{4,0,s}$	$\{5,-76,-68,-63,37\}$	$\{5,-43,-35,-30,-62\}$
$B_{0,1,s}$	$\{1,-57,-74,48,82\}$	$\{1,42,58,15,49\}$
$B_{1,1,s}$	$\{-24,58,81,-17,67\}$	$\{-24,-41,-51,82,34\}$
$B_{2,1,s}$	$\{25,-51,49,-32,9\}$	$\{25,48,-50,67,75\}$
$B_{3,1,s}$	$\{-18,-50,34,42,-8\}$	$\{-18,-17,-65,9,-74\}$
$B_{4,1,s}$	$\{16,-65,75,-41,15\}$	$\{16,-32,-57,-8,81\}$
$B_{0,2,s}$	$\{12,-79,-52,26,-72\}$	$\{12,20,80,-7,60\}$
$B_{1,2,s}$	$\{-46,80,59,-6,78\}$	$\{-46,-19,-73,-72,45\}$
$B_{2,2,s}$	$\{47,-73,60,-21,-13\}$	$\{47,26,-39,78,53\}$
$B_{3,2,s}$	$\{-40,-39,45,20,14\}$	$\{-40,-6,-54,-13,-52\}$
$B_{4,2,s}$	$\{27,-54,53,-19,-7\}$	$\{27,-21,-79,14,59\}$

One can directly check that each $B_{i,j,s}$ sums to zero modulo 165, and that the elements of the blocks of $\mathcal {P}_{s}$, for s∈[0,4], form a half-set of $\mathbb {Z}_{165}\setminus \langle 11\rangle$. Also, since k=5, it is trivial that the partial sums of each block are pairwise distinct. Hence each $\mathcal {P}_s$ is a simple Heffter system in $\mathbb {Z}_{165}$ relative to $\langle 11\rangle$.

We now show that the simple relative Heffter systems constructed in Proposition 3.1 are mutually orthogonal, namely that can be viewed as the parallel classes of a relative Heffter space.

### Theorem 3.3

Let *n* be an odd integer and let *k* be a divisor of *n*. Then there exists a simple cyclic $(nk,k;\Phi (k)+1)_n$ relative Heffter space.

### Proof

Set n=kd, w=n(2k+1) and r=Φ(k)+1, and let $I = \{0\}\cup \textrm{U}(\mathbb {Z}_{k})$. In Proposition 3.1, we constructed *r* simple Heffter systems in $\mathbb {Z}_w$ relative to $\langle 2k+1\rangle$. We denote these relative Heffter systems by $\mathcal {P}_i$ for each i∈I, where each of the $\mathcal {P}_i$’s exactly corresponds to the relative Heffter system denoted in the same way in Proposition 3.1. If we fix *L* to be the same half-set of $\mathbb {Z}_{2k+1}$, then for each $\ell _m \in L$ every relative Heffter system $\mathcal {P}_i$ contains elements of each coset $\ell _m + \langle {2k+1}\rangle$ of the subgroup $\langle {2k+1}\rangle$ of $\mathbb {Z}_w$.

To prove that these Heffter systems are mutually orthogonal, we simply need to prove that for any $t_1, t_2 \in I,$ $t_1\ne t_2$ any block of $\mathcal {P}_{t_1}$ intersects any block of $\mathcal {P}_{t_2}$ in at most one element. Suppose firstly $t_1=0$ and $t_2=s \in \textrm{U}(\mathbb {Z}_{2k+1})$. Note that given two blocks $B_{i_1,j_1,0}\in \mathcal {P}_0$ and $B_{i_2,j_2,s}\in \mathcal {P}_{s}$ with $j_1\ne j_2$ then the elements of the two blocks are contained in different cosets of the subgroup $\langle d(2k+1)\rangle$ which implies $B_{i_1,j_1,0} \cap B_{i_2,j_2,s}=\emptyset$. Hence we can take two blocks of the form $B_{i_1,j,0}\in \mathcal {P}_0$ and $B_{i_2,j,s}\in \mathcal {P}_{s}$, that is:

$$\begin{aligned}\begin{gathered} B_{i_1,j,0}:= \{a_0(i_1d+j)(2k+1)+\ell _0,a_1(i_1d+j)(2k+1)\\ +\ell _1,a_2(i_1d+j)(2k+1)+\ell _2,\ldots ,\\ a_{k-3}(i_1d+j)(2k+1)+\ell _{k-3},{a_{k-2}}(i_1d+j)(2k+1)\\+\ell _{k-2},a_{k-1}(i_1d+j)(2k+1)+\ell _{k-1}\}, \end{gathered}\end{aligned}$$

and

$$\begin{aligned}\begin{gathered} B_{i_2,j,s} = \{a_{i_2}j(2k+1) + \ell _{i_2},a_{1+i_2}(ds+j)(2k+1) \\ + \ell _{1+i_2},a_{2+i_2}(2ds+j)(2k+1)+ \ell _{2+i_2}, \\ \phantom {B_{(i_2,j)} +} \ldots ,a_{k-2+i_2}((i_2-2)ds+j)(2k+1)\\ +\ell _{k-2+i_2},a_{k-1+i_2}((k-1)ds+j)(2k+1)+ \ell _{k-1+i_2}\}. \end{gathered}\end{aligned}$$

By way of contradiction suppose that there exists an $s \in \textrm{U}(\mathbb {Z}_{2k+1})$ such that a block $B_{i_2,j,s}\in \mathcal {P}_{s}$ intersects with a block of $\mathcal {P}_0$ in two distinct elements *x* and *y*. Let then $m_1\in [0, k-1]$ be the index such that $x \equiv \ell _{m_1} \pmod {2k+1}$. Since $x \in B_{i_1,j,0} \cap B_{i_2,j,s}$, the following equation is satisfied:

$$ a_{m_1}(i_1d+j)(2k+1) + \ell _{m_1} = a_{m_1}((m_1-i_2)ds+j)(2k+1) + \ell _{m_1}. $$

This implies:

1 $$\begin{aligned} i_1 = (m_1-i_2)s. \end{aligned}$$

An analogous argument can be carried out for $y \equiv \ell _{m_2} \pmod {2k+1}$ for some $m_2\in [0, k-1]$, $m_2 \ne m_1$, yielding that

2 $$\begin{aligned} i_1 = (m_2-i_2)s. \end{aligned}$$

Since $m_1,m_2\in [0, k-1]$, $m_2 \ne m_1$, and $s \in \textrm{U}(\mathbb {Z}_{k})$, it is clear that (1) and (2) cannot be satisfied at the same time, so we reach a contradiction.

Similarly, assume by contradiction that for two distinct $s_1,s_2 \in \textrm{U}(\mathbb {Z}_{2k+1})$ there exist two blocks $B_{i_1,j_1,s_1} \in \mathcal {P}_{s_1}$ and $B_{i_2,j_2,s_2} \in \mathcal {P}_{s_2}$ intersecting in two distinct elements *x* and *y*. Similarly to the previous case, we can immediately deduce that $j_1=j_2=j$, and if $x \equiv \ell _{m_1} \pmod {2k+1}$ for some $m_1\in [0, k-1]$, then $x \in B_{i_1,j,s_1}\cap B_{i_2,j,s_2}$ implies

$$ a_{m_1}((m_1-i_1)ds_1+j)(2k+1) +\ell _{m_1}= a_{m_1}((m_1-i_2)ds_2+j)(2k+1)+\ell _{m_1}. $$

After some computations, we obtain the following:

3 $$\begin{aligned} (m_1 - i_1)s_1 =(m_1 - i_2)s_2. \end{aligned}$$

Similarly, if $y \equiv \ell _{m_2} \pmod {2k+1}$ for some $m_2\in [0, k-1]$, $m_2 \ne m_1$, then:

$$ a_{m_2}((m_2-i_1)ds_1+j)(2k+1) + \ell _{m_2} = a_{m_2}((m_2-i_2))ds_2+j)(2k+1)+ \ell _{m_2}. $$

We then obtain:

4 $$\begin{aligned} (m_2 - i_1)s_1 =(m_2 -i_2)s_2. \end{aligned}$$

Notice that if we subtract Equation (4) from Equation (3), we obtain the following:

5 $$\begin{aligned} (m_1-m_2)s_1 = (m_1-m_2)s_2. \end{aligned}$$

Since every unit $s \in \textrm{U}(\mathbb {Z}_{2k+1})$ maps each group element $z \in \mathbb {Z}_{2k+1}$ to a unique group element $zs \in \mathbb {Z}_{2k+1}$, it follows that Equation (5) can only hold if $s_1 = s_2$. This is a contradiction.

Moreover, note that since each of the relative Heffter systems is simple, the relative Heffter space is simple. □

### Remark 3.4

When n=k, the above construction produces several relative Heffter spaces with density $\delta \ge 0.7$. In particular, when n=k is prime we obtain a relative Heffter space with $\delta = \frac{n}{n+1}$, which is the densest possible relative Heffter space in a cyclic group (see also Remark 3.11). When $n=k=p^m$ is a prime power, then $\delta = \frac{p^{m-1}(p-1)}{p^m+1}$, which tends to a value of $\delta \ge 0.8$ as $m \rightarrow \infty$. Finally, when n=k=pq is semiprimitive, we obtain a relative Heffter space with $\delta = \frac{(p-1)(q-1)}{pq + 1}$. If p≤q, then we get a relative Heffter space with $\delta \ge 0.7$ for p≥5 and q≥11 or if p,q≥7.

### Example 3.5

One can easily check that the blocks of the relative Heffter systems constructed in Example 3.2 intersect each other in at most one point, meaning that they form a set of 5 mutually orthogonal relative Heffter systems. In other words the blocks of Example 3.2 form a simple cyclic $(75,5;5)_{15}$ relative Heffter space whose five parallel classes are $\mathcal {P}_0,\ldots ,\mathcal {P}_4$.

### Example 3.6

Take n=k=5, then 2k+1=11, v=55, and Φ(k)+1=5. Hence there exists a simple cyclic $(25,5;5)_{5}$ relative Heffter space whose five parallel classes $\mathcal {P}_0,\ldots , \mathcal {P}_4$ are listed below.

**Table:** 

	$\mathcal {P}_0$	$\mathcal {P}_1$	$\mathcal {P}_2$
$B_{0,0,s}$	$\{-10,-2,3,4,5\}$	$\{-10,-24,-8,-7,-6\}$	$\{-10,9,-19,-18,-17\}$
$B_{1,0,s}$	$\{1,-24,25,-18,16\}$	$\{1,9,14,26,5\}$	$\{1,-13,3,15,-6\}$
$B_{2,0,s}$	$\{12,9,-8,15,27\}$	$\{12,-13,-19,4,16\}$	$\{12,20,25,-7,5\}$
$B_{3,0,s}$	$\{23,-13,14,-7,-17\}$	$\{23,20,3,-18,27\}$	$\{23,-2,-8,26,16\}$
$B_{4,0,s}$	$\{-21,20,-19,26,-6\}$	$\{-21,-2,25,15,-17\}$	$\{-21,-24,14,4,27\}$

**Table:** 

	$\mathcal {P}_3$	$\mathcal {P}_4$
$B_{0,0,s}$	$\{-10,-13,25,26,27\}$	$\{-10,20,14,15,16\}$
$B_{1,0,s}$	$\{1,20,-8,4,-17\}$	$\{1,-2,-19,-7,27\}$
$B_{2,0,s}$	$\{12,-2,14,-18,-6\}$	$\{12,-24,3,26,-17\}$
$B_{3,0,s}$	$\{23,-24,-19,15,5\}$	$\{23,9,25,4,-6\}$
$B_{4,0,s}$	$\{-21,9,3,-7,16\}$	$\{-21,-13,-8,-18,5\}$

In the next theorem we present another direct construction of an infinite family of simple cyclic relative Heffter spaces.

### Theorem 3.7

Let p≥3 be a prime and let k∈[3,p]. Then there exists a simple cyclic $(pk,k;p)_{p}$ relative Heffter space.

### Proof

Let *p* and *k* be as in the statement. Let $A = (a_0,\ldots ,a_{k-1})$ be the sequence constructed in Lemma 2.3 (respectively, in Lemma 2.5) if *k* is odd (respectively, if *k* is even). As done in the proof of Proposition 3.1, we can construct an integer half-set $L = \{\ell _0,\ell _1,\ldots ,\ell _{k-1}\}$ of $\mathbb {Z}_{2k+1}$ having a simple ordering.

For any j∈[0,p-1] define $\mathcal {P}_j=\{B_{i,j} \mid i \in [0, p-1]\}$, where

$$ B_{i,j} = \{a_m(i+jm)(2k+1)+\ell _m \mid m\in [0,k-1] \}. $$

As a first remark, we note that each $\mathcal {P}_j$ is a simple Heffter system in $\mathbb {Z}_{p(2k+1)}$ relative to $\langle 2k+1\rangle$. Indeed,

$$ \begin{aligned} \sum B_{i,j}&= \sum _{m=0}^{k-1} ( a_{m} (i+jm)(2k+1)+\ell _{m}) \\&= (2k+1) \left( i \sum _{m=0}^{k-1} a_m+j\sum _{m=0}^{k-1} ma_m \right) + \sum L = 0, \end{aligned} $$

where the last equality holds by Lemma 2.3 if *k* is odd, and by Lemma 2.5 if *k* is even. The simplicity of the blocks of each $\mathcal {P}_j$ and the fact that they partition a half-set of $\mathbb {Z}_{p(2k+1)} \setminus \langle 2k+1\rangle$ follows by an argument analogous to the one of Proposition 3.1.

To verify that $\{\mathcal {P}_j \mid j \in [0,p-1]\}$ is a set of mutually orthogonal relative Heffter systems, assume by way of contradiction that there exist two distinct blocks $B_{i_1,j_1}$ and $B_{i_2,j_2}$ having two common elements *x* and *y*. Then let $m_1\in [0,k-1]$ be the index such that $x \equiv \ell _{m_1} \pmod {2k+1}$. Since $x \in B_{i_1,j_1}\cap B_{i_2,j_2}$, the following equation holds:

$$ a_{m_1} (i_1+j_1m_1) (2k+1) + \ell _{m_1} = a_{m_1} (i_2+j_2m_1) (2k+1) + \ell _{m_1} $$

That implies:

6 $$\begin{aligned} i_1+j_1m_1 = i_2+j_2m_1. \end{aligned}$$

Similarly, if $y \equiv \ell _{m_2} \pmod {2k+1}$, then $y \in B_{i_1,j_1}\cap B_{i_2,j_2}$ implies:

7 $$\begin{aligned} i_1+j_1m_2 = i_2+j_2m_2. \end{aligned}$$

If we subtract Equation (7) from Equation (6) we obtain the following:

$$ j_1(m_1-m_2) = j_2(m_1-m_2), $$

that implies $j_1 = j_2$, hence $i_1=i_2$. This is a contradiction, hence $\{\mathcal {P}_j \mid j \in [0,p-1]\}$ is a simple cyclic $(pk,k;p)_{p}$ relative Heffter space. □

### Example 3.8

Take p=7 and k=6, then $\mathcal {P}_0, \dotsc , \mathcal {P}_6$ listed below are the parallel classes of a simple cyclic $(42,6;7)_{7}$ relative Heffter space:

**Table:** 

	$\mathcal {P}_0$	$\mathcal {P}_1$	$\mathcal {P}_2$
$B_{0,j}$	$\{ 7 , 1 , -2 , 3 , -4 , -5\}$	$\{ 7 , 27 , 24 , -36 , -43 , 21\}$	$\{ 7 , -38 , -41 , 16 , 9 , -44\}$
$B_{1,j}$	$\{ -19 , 27 , 11 , -10 , 9 , -18\}$	$\{ -19 , -38 , 37 , 42 , -30 , 8\}$	$\{ -19 , -12 , -28 , 3 , 22 , 34\}$
$B_{2,j}$	$\{ -45 , -38 , 24 , -23 , 22 , -31\}$	$\{ -45 , -12 , -41 , 29 , -17 , -5\}$	$\{ -45 , 14 , -15 , -10 , 35 , 21\}$
$B_{3,j}$	$\{ 20 , -12 , 37 , -36 , 35 , -44\}$	$\{ 20 , 14 , -28 , 16 , -4 , -18\}$	$\{ 20 , 40 , -2 , -23 , -43 , 8\}$
$B_{4,j}$	$\{ -6 , 14 , -41 , 42 , -43 , 34\}$	$\{ -6 , 40 , -15 , 3 , 9 , -31\}$	$\{ -6 , -25 , 11 , -36 , -30 , -5\}$
$B_{5,j}$	$\{ -32 , 40 , -28 , 29 , -30 , 21\}$	$\{ -32 , -25 , -2 , -10 , 22 , -44\}$	$\{ -32 , 1 , 24 , 42 , -17 , -18\}$
$B_{6,j}$	$\{ 33 , -25 , -15 , 16 , -17 , 8\}$	$\{ 33 , 1 , 11 , -23 , 35 , 34\}$	$\{ 33 , 27 , 37 , 29 , -4 , -31\}$

**Table:** 

	$\mathcal {P}_3$	$\mathcal {P}_4$	$\mathcal {P}_5$
$B_{0,j}$	$\{ 7 , -12 , -15 , -23 , -30 , -18\}$	$\{ 7 , 14 , 11 , 29 , 22 , 8\}$	$\{ 7 , 40 , 37 , -10 , -17 , 34\}$
$B_{1,j}$	$\{ -19 , 14 , -2 , -36 , -17 , -31\}$	$\{ -19 , 40 , 24 , 16 , 35 , -5\}$	$\{ -19 , -25 , -41 , -23 , -4 , 21\}$
$B_{2,j}$	$\{ -45 , 40 , 11 , 42 , -4 , -44\}$	$\{ -45 , -25 , 37 , 3 , -43 , -18\}$	$\{ -45 , 1 , -28 , -36 , 9 , 8\}$
$B_{3,j}$	$\{ 20 , -25 , 24 , 29 , 9 , 34\}$	$\{ 20 , 1 , -41 , -10 , -30 , -31\}$	$\{ 20 , 27 , -15 , 42 , 22 , -5\}$
$B_{4,j}$	$\{ -6 , 1 , 37 , 16 , 22 , 21\}$	$\{ -6 , 27 , -28 , -23 , -17 , -44\}$	$\{ -6 , -38 , -2 , 29 , 35 , -18\}$
$B_{5,j}$	$\{ -32 , 27 , -41 , 3 , 35 , 8\}$	$\{ -32 , -38 , -15 , -36 , -4 , 34\}$	$\{ -32 , -12 , 11 , 16 , -43 , -31\}$
$B_{6,j}$	$\{ 33 , -38 , -28 , -10 , -43 , -5\}$	$\{ 33 , -12 , -2 , 42 , 9 , 21\}$	$\{ 33 , 14 , 24 , 3 , -30 , -44\}$

**Table:** 

	$$\mathcal {P}_6$$
$B_{0,j}$	$\{ 7 , -25 , -28 , 42 , 35 , -31\}$
$B_{1,j}$	$\{ -19 , 1 , -15 , 29 , -43 , -44\}$
$B_{2,j}$	$\{ -45 , 27 , -2 , 16 , -30 , 34\}$
$B_{3,j}$	$\{ 20 , -38 , 11 , 3 , -17 , 21\}$
$B_{4,j}$	$\{ -6 , -12 , 24 , -10 , -4 , 8\}$
$B_{5,j}$	$\{ -32 , 14 , 37 , -23 , 9 , -5\}$
$B_{6,j}$	$\{ 33 , 40 , -41 , -36 , 22 , -18\}$

In the last part of this section we focus on the special case in which the block size equals the degree of the space (and hence the number of points and blocks are equal). Note that the following is a consequence both of Theorem 3.3 and Theorem 3.7.

### Corollary 3.9

Given a prime p≥3, then there exists a simple cyclic $(p^2,p;p)_{p}$ relative Heffter space.

In the case of the above corollary we have a resolvable configuration where the number of points is the square of the block size, hence we find again the concept of a *net*. So, following the terminology introduced in [8], we call the relative Heffter space of Corollary 3.9 a relative Heffter net. The densest Heffter net known so far has been obtained, with the aid of a computer, in [8] and it has parameters (121, 11; 9) and hence density δ=0.75. The relative Heffter net constructed in Corollary 3.9 has density $\delta =\frac{p}{p+1}$, which asymptotically approaches 1. We recall that in [8] (see Corollary 2.2) the authors proved that a linear cyclic Heffter space cannot exists, namely that it is not possible, working in a cyclic group, to construct a Heffter space with density one. Since the proof of this result also holds in the more general case where the point set is a half-set of $\mathbb {Z}_w\setminus J$ and *J* is not necessarily the trivial subgroup, we can state the following.

### Proposition 3.10

A cyclic relative Heffter space cannot be linear.

This allows us state the make the following consideration.

### Remark 3.11

For any prime p≥3, the $(p^2,p;p)_{p}$ relative Heffter net of Corollary 3.9 is the densest Heffter net which can be constructed on $\mathbb {Z}_{p(2p+1)}$ relative to $\langle 2p+1 \rangle$.

## Globally simple relative Heffter arrays

As explained in the Introduction, a relative Heffter space of degree two is completely equivalent to a relative Heffter array. This means that, as a consequence of the results obtained in the previous section, we get new constructions for relative Heffter arrays. Moreover, these arrays satisfy the very strong additional condition of being *globally simple*, a property introduced in [16].

As usual, with a little abuse of notation, we can identify each row (respectively, column) of a (relative) Heffter array $\textrm{H}_t(n; k)$ with the set of size *k* whose elements are the entries of the nonempty cells of such a row (respectively, column). A (relative) Heffter array is *simple* if each row and each column admit a simple ordering. Hence, to verify this property we need to provide an ordering for each row and each column which is simple. Clearly, larger *n* and *k* are longer and more tedious is to provide explicit simple orderings for rows and columns of an $\textrm{H}_t(n;k)$. For this reason, in [16] the authors introduced the concept of a *globally simple* Heffter array, namely a Heffter array which is simple with respect to the natural ordering of rows (namely from left to right) and columns (namely from top to bottom). It is evident that to construct globally simple (relative) Heffter arrays is much more difficult than to construct simple (relative) Heffter arrays. Infinite classes of globally simple Heffter arrays can be found in [4, 10, 13, 15, 18, 22]. At the moment, as far as we know, there are only two classes of globally simple relative Heffter arrays, that are $\textrm{H}_7(n;7)$ and $\textrm{H}_9(n;9)$, constructed in [17]. Hence in the following, we present the first two infinite classes of globally simple relative Heffter arrays in which the block size is not fixed.

### Theorem 4.1

There exists a globally simple $\textrm{H}_n(n;k)$ for every odd n≥3 and every *k* dividing *n*.

### Proof

Let n=kd be odd. Let $\mathcal {H} = \{\mathcal {P}_s \mid s=0 \text { or } \gcd (s,k)=1\}$ be the $(nk,k;\Phi (k)+1)_n$ relative Heffter space of Theorem 3.3, and consider the two relative Heffter systems $\mathcal {P}_0$ and $\mathcal {P}_{1}$. Construct the n×n array *H* such that for every g,h∈[0,d-1] and i,j∈[0,k-1] the (gk+i+1,hk+j+1)-th cell of *H* is filled with the element belonging to $B_{i,g,0}\cap B_{j,h,1}$ if it exists, and it is empty otherwise, where we recall that $B_{i,g,0} \in \mathcal {P}_0$ and $B_{j,h,1}\in \mathcal {P}_1$. Clearly, the array *H* is an $\textrm{H}_n(n;k)$; in what follows, we show that it is globally simple.

Assume that two blocks $B_{i,g,0}$ and $B_{j,h,1}$ share a common element *x*. We recall that for every g,h∈[0,d-1] and i,j∈[0,k-1]:

$$\begin{aligned}\begin{gathered} B_{i,g,0} = \{a_m(id+g)(2k+1)+\ell _m \mid m \in [0,k-1]\}, \end{gathered}\end{aligned}$$

$$\begin{aligned}\begin{gathered} B_{j,h,1} = \{a_{m+j}(md+h)(2k+1)+\ell _{m+j}\mid m \in [0,k-1]\}. \end{gathered}\end{aligned}$$

We have $x \in B_{i,g,0} \cap B_{j,h,1}$, with $x \equiv \ell _{m_1} \pmod {2k+1} \equiv \ell _{m_2+j} \pmod {2k+1}$ for some $m_1,m_2 \in [0,k-1]$, hence:

$$ a_{m_1}(id+g)(2k+1)+\ell _{m_1} \equiv a_{m_2+j}(m_2d+h)(2k+1)+\ell _{m_2+j} \pmod {n(2k+1)}. $$

Clearly, it follows that $m_1 \equiv m_2 + j \pmod {2k+1}$, thus $\ell _{m_1} = \ell _{m_2+j}$ and $a_{m_1}=a_{m_2+j}$. From the previous equation, we derive that $id+g \equiv m_2d+h \pmod {n}$; since *d* divides *n*, it can easily be seen that necessarily g=h, hence $i=m_2$. As a first consequence, we have shown that $B_{i,g,0} \cap B_{j,h,1}$ is nonempty if and only if g=h. Moreover, as $i=m_2$, we immediately derive that the (gk+j+1)-th column of *H* is filled with the sequence:

$$ (a_{j}g(2k+1) + \ell _{j},a_{1+j}(d+g)(2k+1) + \ell _{1+j}, \ldots ,a_{k-1+j}((k-1)d+g)(2k+1)+ \ell _{k-1+j}), $$

that is simple by construction (see Proposition 3.1). Finally, from $m_1 \equiv m_2 + j \pmod {2k+1}$ and $i=m_2$ it can be seen that the (gk+i+1)-th row of *H* is filled with the sequence:

$$ (a_{i}(id+j)(2k+1)+\ell _{i}, a_{i+1}(id+j)(2k+1)+\ell _{i+1}, \dotsc , a_{i+k-1}(id+j)(2k+1)+\ell _{i+k-1}), $$

that is a cyclic permutation of the ordering

$$ (a_{0}(id+j)(2k+1)+\ell _{0},a_{1}(id+j)(2k+1)+\ell _{1},\dotsc , a_{k-1}(id+j)(2k+1)+\ell _{k-1} ), $$

that is simple by construction (see again Proposition 3.1). Hence, the rows and columns of *H* are simple with respect to their natural ordering, and the array *H* is a globally simple $\textrm{H}_n(n;k)$. □

### Example 4.2

The following is a globally simple $\textrm{H}_{15}(15;5)$ whose rows (columns, respectively) correspond to the blocks of the Heffter system $\mathcal {P}_0$ ($\mathcal {P}_1$, respectively) constructed in Example 3.2. We recall that the entries of the array are elements in $\mathbb {Z}_{165}$.

$$ \begin{array}{|c|c|c|c|l|c|c|c|c|c|c|c|c|c|c|}\hline -10 &  -2 &  3 &  4 &  5 &  &  &  &  &  &  &  &  &  &  \\ \hline -68 &  69 &  -62 &  38 &  23 &  &  &  &  &  &  &  &  &  &  \\ \hline -30 &  37 &  71 &  56 &  31 &  &  &  &  &  &  &  &  &  &  \\ \hline -29 &  -61 &  -76 &  -35 &  36 &  &  &  &  &  &  &  &  &  &  \\ \hline -28 &  -43 &  64 &  -63 &  70 &  &  &  &  &  &  &  &  &  &  \\ \hline &  &  &  &  &  1 &  -24 &  25 &  -18 &  16 &  &  &  &  &  \\ \hline &  &  &  &  &  75 &  -74 &  81 &  49 &  34 &  &  &  &  &  \\ \hline &  &  &  &  &  -8 &  15 &  82 &  67 &  9 &  &  &  &  &  \\ \hline &  &  &  &  &  -51 &  -50 &  -65 &  -57 &  58 &  &  &  &  &  \\ \hline &  &  &  &  &  -17 &  -32 &  42 &  -41 &  48 &  &  &  &  &  \\ \hline &  &  &  &  &  &  &  &  &  &  12 &  -46 &  47 &  -40 &  27\\ \hline &  &  &  &  &  &  &  &  &  &  53 &  -52 &  59 &  60 &  45\\ \hline &  &  &  &  &  &  &  &  &  &  14 &  -7 &  -72 &  78 &  -13\\ \hline &  &  &  &  &  &  &  &  &  &  -73 &  -39 &  -54 &  -79 &  80\\ \hline &  &  &  &  &  &  &  &  &  &  -6 &  -21 &  20 &  -19 &  26\\ \hline \end{array} $$

The arrays constructed in Theorem 4.1 have a block-diagonal structure, as shown in Example 4.2, while the arrays we are going to construct in the next theorem have a diagonal structure, so it is convenient to introduce the following notation. If *H* is an n×n array, for i∈[1,n] we define the *i*-th diagonal

$$\begin{aligned} D_i=\{(i,1),(i+1,2),\ldots ,(i-1,n)\}. \end{aligned}$$

Here all arithmetic on the row and the column indices is performed modulo *n*, where the set of reduced residues is $\{1,2,\ldots ,n\}$. We say that the diagonals $D_i,D_{i+1},\ldots , D_{i+r}$ are *consecutive diagonals*.

### Definition 4.3

Let k≥1 be an integer. A square Heffter array *H* of size n≥k is *cyclically* *k*-*diagonal* if the nonempty cells of *H* are exactly those of *k* consecutive diagonals.

### Theorem 4.4

There exists a cyclically *k*-diagonal globally simple $\textrm{H}_p(p;k)$ for every prime p≥3 and every k∈[3,p].

### Proof

Let *p* and *k* be as in the statement. Let $\mathcal {H} = \{\mathcal {P}_s \mid s \in [0,p-1]\}$ be the $(pk,k;p)_p$ relative Heffter space of Theorem 3.7, and consider the two relative Heffter systems $\mathcal {P}_0$ and $\mathcal {P}_{p-1}$. Construct the p×p partially filled array *H* whose (*i*, *j*)-th cell contains the element belonging to $B_{i-1,0} \cap B_{j-1,p-1}$ if it exists, and it is empty otherwise. Clearly, *H* is an $\textrm{H}_p(p;k)$; in what follows, we show that it is cyclically *k*-diagonal and globally simple.

Assume that two blocks $B_{i-1,0} \in \mathcal {P}_0$ and $B_{j-1,p-1}\in \mathcal {P}_{p-1}$ share a common element *x*. We recall that for each i,j∈[0,p-1],

$$ \begin{aligned} B_{i-1,0}&= \{a_m(i-1)(2k+1)+ \ell _m \mid m\in [0,k-1]\} \\ B_{j-1,p-1}&= \{a_m(j-1+m(p-1))(2k+1)+\ell _m \mid m\in [0,k-1]\}, \end{aligned} $$

where $A = (a_0,\dotsc , a_{k-1})$ is the sequence of Lemma 2.3 if *k* is odd, and of Lemma 2.5 if *k* is even, and $L = \{\ell _0, \dotsc , \ell _{k-1}\}$ is an integer half-set of $\mathbb {Z}_{2k+1}$ having a simple ordering $(\ell _0,\dotsc , \ell _{k-1})$, see Proposition 2.1.

From the expression of $x \in B_{i-1,0} \cap B_{j-1,n-1}$, with $x \equiv \ell _m \pmod {2k+1}$, we obtain the following equation:

$$ a_m (i-1)(2k+1)+ \ell _m \equiv a_m (j-1+m(p-1))(2k+1)+ \ell _m \pmod {p(2k+1)}, $$

that implies $i \equiv j+m(p-1) \pmod {p}$, hence $m \equiv j-i \pmod {p}$. Note that from this equivalence we deduce that the (*i*, *j*)-th cell of *H* is filled if and only if $j-i \pmod {p} \in [0, k-1]$, hence *H* is cyclically *k*-diagonal. It then follows that the *i*-th row of *H* read with respect to its natural ordering is a cyclic permutation of the ordering

$$\begin{aligned}  &   (a_0 (i-1) (2k+1)+\ell _0,a_1 (i-1)(2k+1) + \ell _1, \dotsc , a_{k-2} (i-1)(2k+1)\\    &   \qquad +\ell _{k-2},a_{k-1} (i-1)(2k+1)+\ell _{k-1}). \end{aligned}$$

From Theorem 3.7 we have that ω is a simple ordering, and since a cyclic permutation of a simple ordering of a zero-sum set is simple as well, the *i*-th row of *H* read with respect to its natural ordering is simple. Similarly, it can be seen that the elements contained in the *j*-th column of *H* read with respect to its natural ordering is a cyclic permutation of the ordering

$$ \begin{aligned} (&a_{k-1} (j+(p-1)(k-1))(2k+1)+\ell _{k-1}, a_{k-2} (j+(p-1)(k-2))(2k+1)+\ell _{k-2}, \dotsc , \\  &a_{1} (j+p-1)(2k+1)+\ell _{1}, a_{0} j(2k+1)+\ell _{0}). \end{aligned} $$

Therefore, also each column of *H* is simple with respect to natural ordering. We can conclude that *H* is a cyclically *k*-diagonal globally simple $\textrm{H}_p(p;k)$. □

### Remark 4.5

In Theorems 4.1 and 4.4 we obtain the arrays starting from two particular parallel classes of the relative Heffter space constructed in Theorems 3.3 and 3.7, respectively. Reasoning in the same way, it can be shown that a globally simple relative Heffter array can be constructed from each pair of distinct parallel classes.

### Example 4.6

The following is a globally simple cyclically 6-diagonal $\textrm{H}_7(7;6)$ whose rows (respectively, columns) correspond to the blocks of the relative Heffter system $\mathcal {P}_0$ (respectively, $\mathcal {P}_6$) constructed in Example 3.8. We recall that the entries of the array are elements of $\mathbb {Z}_{91}$.

$$ \begin{array}{|r|r|r|r|r|r|r|} \hline 7 &  1 &  -2 &  3 &  -4 &  -5 &  \\ \hline &  -19 &  27 &  11 &  -10 &  9 &  -18\\ \hline -31 &  &  -45 &  -38 &  24 &  -23 &  22\\ \hline 35 &  -44 &  &  20 &  -12 &  37 &  -36\\ \hline 42 &  -43 &  34 &  &  -6 &  14 &  -41\\ \hline -28 &  29 &  -30 &  21 &  &  -32 &  40\\ \hline -25 &  -15 &  16 &  -17 &  8 &  &  33\\ \hline \end{array} $$

## Orthogonal cycle decompositions and biembeddings

It is well known that Heffter arrays give rise to graph decompositions obtainable via difference methods (see Section 5 of [26] for details). More generally, in [8] the authors use Heffter spaces to construct sets of mutually orthogonal cycle systems, and the same can also be done starting from *relative* Heffter spaces. To explain this we firstly introduce some background on this topic. By $K_{m\times n}$ we denote the complete multipartite graph with *m* parts of size *n*, and we recall that a *k*-*cycle decomposition* of $K_{m\times n}$ is a set of *k*-cycles whose edges partition the edge-set of $K_{m\times n}$. Such a decomposition $\mathcal {D}$ is said to be *G*-*regular* if the vertex set of $K_{m\times n}$ is an additive group *G* and $C+g \in \mathcal {D}$ for every pair $(C,g)\in \mathcal {D} \times G$. Equivalently if, up to isomorphism, *G* is an automorphism group of $\mathcal {D}$. If *G* is a cyclic group one simply speaks of a *cyclic* decomposition. Two cycle decompositions, say $\mathcal {D}$ and ${\mathcal {D}}'$, of a graph *K* are *orthogonal* if there is no cycle of $\mathcal {D}$ sharing more than one edge with a cycle of ${\mathcal {D}}'$.

The construction of a set of mutually orthogonal cycle decompositions of the complete graph was recently considered in [7–9, 20], but to our knowledge, there are no results on sets of size greater than two of mutually orthogonal cycle decompositions of the complete multipartite graph. On the other hand, if there exists a simple relative Heffter array, then there exist two orthogonal cycle decompositions of the complete multipartite graph. To explain this we have to introduce some notation. Given an n×n partially filled array *H*, we denote by $\mathcal {E}(H)$ the set of the elements of the filled cells of *H*. Analogously, by $\mathcal {E}(R_i)$ and $\mathcal {E}(C_j)$ we mean the elements of the *i*-th row and of the *j*-th column, respectively, of *H*. Also, by $\omega _{R_i}$ and $\omega _{C_j}$ we denote, respectively, an ordering of $\mathcal {E}(R_i)$ and of $\mathcal {E}(C_j)$. If for any i,j∈[1,n], the orderings $\omega _{R_i}$ and $\omega _{C_j}$ are simple, we denote by $\omega _r=\omega _{R_1}\circ \ldots \circ \omega _{R_n}$ the simple ordering for the rows and by $\omega _c=\omega _{C_1}\circ \ldots \circ \omega _{C_n}$ the simple ordering for the columns. The relationship between simple relative Heffter arrays and cyclic cycle decompositions of the complete multipartite graph is explained in detail in [15]. Here we briefly recall the following result.

### Proposition 5.1

[15, Proposition 2.9] Let *H* be a simple $\textrm{H}_t(n;k)$ with respect to the orderings $\omega _r$ and $\omega _c$. Then there exist two cyclic *k*-cycle decompositions $\mathcal {D}_{\omega _r}$ and $\mathcal {D}_{\omega _c}$ of $K_{\frac{2nk+t}{t}\times t}$. Moreover the decompositions $\mathcal {D}_{\omega _r}$ and $\mathcal {D}_{\omega _c}$ are orthogonal.

Since the relative Heffter spaces constructed in Sect. 3 are simple, here we obtain, as a consequence, sets with many mutually orthogonal cycle decompositions of the complete multipartite graph. In fact, it is not hard to see that the following proposition holds.

### Proposition 5.2

If there exists a simple $(nk, k;r)_t$ relative Heffter space over *G*, then there exist *r* mutually orthogonal *G*-regular *k*-cycle decompositions of $K_{\frac{2nk+t}{t}\times t}$.

The above proposition is nothing but a generalization of Proposition 5.1 and the proof can be obtained reasoning in the same way. This connection between simple relative Heffter spaces and cycle decompositions allows us to state the following results.

### Theorem 5.3

Let *n* be an odd integer and *k* be a divisor of *n*. Then there exist at least Φ(k)+1 mutually orthogonal cyclic *k*-cycle decompositions of $K_{(2k+1)\times n}$.

### Proof

The result follows by Theorem 3.3 and Proposition 5.2. □

### Theorem 5.4

For every prime p≥3 and k∈[3,p], there exist at least *p* mutually orthogonal cyclic *k*-cycle decompositions of $K_{(2k+1)\times p}$.

### Proof

The result follows by Theorem 3.7 and Proposition 5.2. □

Since the decompositions so constructed are cyclic, to describe them it is sufficient to give a set of the so- called *base blocks*, then all the other cycles of the decompositions can be obtained considering the orbit of the base blocks under the natural action of the cyclic group.

### Example 5.5

Starting from the blocks of the seven parallel classes of the simple relative Heffter space presented in Example 3.8, we construct the base blocks of seven mutually orthogonal cyclic 6-cycle decompositions of $K_{13 \times 7}$ under the action of the group $\mathbb {Z}_{91}$. In the following, let $\mathcal {D}_j$ denote the decomposition obtained from the parallel class $\mathcal {P}_j$, where j∈[0,6]. In detail, let $C_{i,j}$ be the graph whose vertices are the ordered partial sums of $B_{i,j}$, for each i,j∈[0,6]. Since each block $B_{i,j}$ has size 6, sums to zero, and the ordering is simple, the resulting graph $C_{i,j}$ is a 6-cycle. In particular we denote the vertices of the cycles with an element in the range [-45,45], since, in this way, it is easier to check that the vertices are actually pairwise distinct.

**Table:** 

	$\mathcal {D}_0$	$\mathcal {D}_1$	$\mathcal {D}_2$
$C_{0,j}$	(7, 8, 6, 9, 5, 0)	(7,34,-33,22,-21,0)	(7,-31,19,35,44,0)
$C_{1,j}$	(-19,8,19,9,18,0)	(-19,34,-20,22,-8,0)	(-19,-31,32,35,-34,0)
$C_{2,j}$	(-45,8,32,9,31,0)	(-45,34,-7,22,5,0)	(-45,-31,45,35,-21,0)
$C_{3,j}$	(20, 8, 45, 9, 44, 0)	(20, 34, 6, 22, 18, 0)	(20,-31,-33,35,-8,0)
$C_{4,j}$	(-6,8,-33,9,-34,0)	(-6,34,19,22,31,0)	(-6,-31,-20,35,5,0)
$C_{5,j}$	(-32,8,-20,9,-21,0)	(-32,34,32,22,44,0)	(-32,-31,-7,35,18,0)
$C_{6,j}$	(33,8,-7,9,-8,0)	(33,34,45,22,-34,0)	(33,-31,6,35,31,0)

**Table:** 

	$\mathcal {D}_3$	$\mathcal {D}_4$	$\mathcal {D}_5$
$C_{0,j}$	(7,-5,-20,-43,18,0)	(7,21,32,-30,-8,0)	(7,-44,-7,-17,-34,0)
$C_{1,j}$	(-19,-5,-7,-43,31,0)	(-19,21,45,-30,5,0)	(-19,-44,6,-17,-21,0)
$C_{2,j}$	(-45,-5,6,-43,44,0)	(-45,21,-33,-30,18,0)	(-45,-44,19,-17,-8,0)
$C_{3,j}$	(20,-5,19,-43,-34,0)	(20,21,-20,-30,31,0)	(20,-44,32,-17,5,0)
$C_{4,j}$	(-6,-5,32,-43,-21,0)	(-6,21,-7,-30,44,0)	(-6,-44,-45,-17,18,0)
$C_{5,j}$	(-32,-5,45,-43,-8,0)	(-32,21,6,-30,-34,0)	(-32,-44,-33,-17,31,0)
$C_{6,j}$	(33,-5,-33,-43,5,0)	(33,21,19,-30,-21,0)	(33,-44,-20,-17,44,0)

**Table:** 

	$$\mathcal {D}_6$$
$C_{0,j}$	(7,-18,-46,-4,31,0)
$C_{1,j}$	(-19,-18,-33,-4,-44,0)
$C_{2,j}$	(-45,-18,-20,-4,-34,0)
$C_{3,j}$	(20,-18,-7,-4,-21,0)
$C_{4,j}$	(-6,-18,6,-4,-8,0)
$C_{5,j}$	(-32,18,19,-4,5,0)
$C_{6,j}$	(33,-18,32,-4,18,0)

We conclude this section by showing that, as a consequence of results in Section 4, we can obtain new results concerning biembeddings of cycle decompositions. Actually, in [1], one of Archdeacon’s main motivations for defining Heffter arrays was due to their applications, in particular, owing to their usefulness in identifying biembeddings of cycle decompositions. Then in [17], generalizing some of Archdeacon’s results, the authors showed how starting from a relative Heffter array it is also possible to obtain suitable biembeddings. Firstly, we need to recall some definitions and results, we start from the following definition, see [23].

### Definition 5.6

An *embedding* of a graph Γ in a surface Σ is a continuous injective mapping $\psi : \Gamma \rightarrow \Sigma$, where Γ is viewed with the usual topology as 1-dimensional simplical complex.

The connected components of $\Sigma \setminus \psi (\Gamma )$ are called ψ-*faces*. If each ψ-face is homeomorphic to an open disk, then the embedding ψ is said to be *cellular*.

### Definition 5.7

A *biembedding* of two cycle decompositions $\mathcal {D}$ and $\mathcal {D}'$ of a simple graph Γ is a face 2-colorable embedding of Γ in which one color class is comprised of the cycles in $\mathcal {D}$ and the other class contains the cycles in $\mathcal {D}'$.

Given a relative Heffter array $H=\textrm{H}_t(n;k)$, the orderings $\omega _r$ and $\omega _c$ are said to be *compatible* if $\omega _c \circ \omega _r$ is a cycle of length $\mathcal {E}(H)$. The connection between relative Heffter arrays and biembeddings has been established in [17] with the following result.

### Theorem 5.8

[17, Theorem 3.4] Let *H* be a relative Heffter array $\textrm{H}_t(n;k)$ that is simple with respect to the compatible orderings $\omega _r$ and $\omega _c$. Then there exists a cellular biembedding of the cyclic *k*-cycle decompositions $\mathcal {D}_{\omega _r^{-1}}$ and $\mathcal {D}_{\omega _c}$ of $K_{\frac{2nk+t}{t}\times t}$ into an orientable surface of genus

$$\begin{aligned} g=1+\frac{(nk-2n-1)(2nk+t)}{2}. \end{aligned}$$

The arrays constructed in the previous section are not only simple, but they are globally simple. Looking for compatible orderings in the case of a globally simple Heffter array led to investigate the following problem introduced in [14]. Let *A* be an m×n toroidal partially filled array. By $r_i$ we denote the orientation of the *i*-th row, precisely $r_i=1$ if it is from left to right and $r_i=-1$ if it is from right to left. Analogously, for the *j*-th column, if its orientation $c_j$ is from top to bottom then $c_j=1$ otherwise $c_j=-1$. Assume that an orientation $\mathcal {R}=(r_1,\dots ,r_m)$ and $\mathcal {C}=(c_1,\dots ,c_n)$ is fixed. Given an initial filled cell $(i_1,j_1)$ consider the sequence $L_{\mathcal {R},\mathcal {C}}(i_1,j_1)=((i_1,j_1),(i_2,j_2),\ldots ,(i_\ell ,j_\ell ),$ $(i_{\ell +1},j_{\ell +1}),\ldots )$ where $j_{\ell +1}$ is the column index of the filled cell $(i_\ell ,j_{\ell +1})$ of the row $R_{i_\ell }$ next to $(i_\ell ,j_\ell )$ in the orientation $r_{i_\ell }$, and where $i_{\ell +1}$ is the row index of the filled cell of the column $C_{j_{\ell +1}}$ next to $(i_\ell ,j_{\ell +1})$ in the orientation $c_{j_{\ell +1}}$. The problem is the following:

### Crazy Knight’s Tour Problem

Given a toroidal partially filled array *H*, do there exist $\mathcal {R}$ and $\mathcal {C}$ such that the list $L_{\mathcal {R},\mathcal {C}}$ covers all the filled cells of *H*?

By *P*(*H*) we will denote the Crazy Knight’s Tour Problem for a given array *H*. Also, given a filled cell (*i*, *j*), if $L_{\mathcal {R},\mathcal {C}}(i,j)$ covers all the filled positions of *H* we will say that $(\mathcal {R},\mathcal {C})$ is a solution of *P*(*H*). In [14, 16, 21] it is proved that *P*(*H*) admits a solution for several classes of (not necessarily square) arrays, here we recall only the following result we need for the purpose of this paper.

### Proposition 5.9

[16, Proposition 3.4] Let k≥3 be an odd integer and let *H* be a cyclically *k*-diagonal array of size n>k. If gcd(n;k-1)=1, then *P*(*H*) admits a solution.

The relationship between the Crazy Knight’s Tour Problem and globally simple relative Heffter arrays is explained in the following result which is an easy consequence of Theorem 5.8.

### Corollary 5.10

[17, Corollary 3.5] Let *H* be a globally simple relative Heffter array $\textrm{H}_t(n;k)$ such that *P*(*H*) admits a solution $(\mathcal {R},\mathcal {C})$. Then there exists a biembedding of the cyclic cycle decompositions $\mathcal {D}_{\omega _r^{-1}}$ and $\mathcal {D}_{\omega _c}$ of $K_{\frac{2nk+t}{t}\times t}$ into an orientable surface.

Our main result about biembeddings is the following.

### Theorem 5.11

For every prime p≥3 and every k∈[3,p], there exists a biembedding of the cyclic *k*-cycle decompositions $\mathcal {D}_{\omega _r^{-1}}$ and $\mathcal {D}_{\omega _c}$ of $K_{(2k+1)\times p}$ into an orientable surface.

### Proof

The result follows from Theorem 4.4, Proposition 5.9 and Corollary 5.10. □
